# Thermal Decoupling May Promote Cooling and Avoid Heat Stress in Alpine Plants

**DOI:** 10.3390/plants14132023

**Published:** 2025-07-02

**Authors:** Loreto V. Morales, Angela Sierra-Almeida, Catalina Sandoval-Urzúa, Mary T. K. Arroyo

**Affiliations:** 1 Grupo de Ecofisiología Térmica (GET), Facultad de Ciencias Naturales y Oceanográficas, Universidad de Concepción, Concepción 4030000, Chile; loretomorales@udec.cl (L.V.M.); or caatalinaasandoval@gmail.com (C.S.-U.); 2 Cape Horn International Center (CHIC), Cabo de Hornos 6350000, Chile; southern@uchile.cl; 3 Departamento de Ciencias Ecológicas, Facultad de Ciencias, Universidad de Chile, Ñuñoa 7800003, Chile; 4 Instituto de Ecología y Biodiversidad (IEB), Concepción 4030000, Chile

**Keywords:** alpine, heat resistance, microclimate, short plants, thermal decoupling

## Abstract

In alpine ecosystems, where low temperatures predominate, prostrate growth forms play a crucial role in thermal resistance by enabling thermal decoupling from ambient conditions, thereby creating a warmer microclimate. However, this strategy may be maladaptive during frequent heatwaves driven by climate change. This study combined microclimatic and plant characterization, infrared thermal imaging, and leaf photoinactivation to evaluate how thermal decoupling (TD) affects heat resistance (LT_50_) in six alpine species from the Nevados de Chillán volcano complex in the Andes of south-central Chile. Results showed that plants’ temperatures increased with solar radiation, air, and soil temperatures, but decreased with increasing humidity. Most species exhibited negative TD, remaining 6.7 K cooler than the air temperature, with variation across species, time of day, and growth form; shorter, rounded plants showed stronger negative TD. Notably, despite negative TD, all species exhibited high heat resistance (Mean LT_50_ = 46 °C), with LT_50_ positively correlated with TD in shrubs. These findings highlight the intricate relationships between thermal decoupling, environmental factors, and plant traits in shaping heat resistance. This study provides insights into how alpine plants may respond to the increasing heat stress associated with climate change, emphasizing the adaptive significance of thermal decoupling in these environments.

## 1. Introduction

Temperature is a major environmental driver of plant distribution and diversity across various ecosystems, and its influence is particularly pronounced in alpine habitats due to their unique climatic conditions [1]. Temperature plays a critical role in all plant life processes, such as plant metabolism and energy balance, including membrane properties, enzyme activity, the rate of chemical reactions and diffusion, and physical processes such as transpiration and the volatilization of specific compounds [2,3,4,5,6]. Temperature also controls the pattern and timing of plant development and reproduction, accounting for phenological processes such as the timing of germination and flowering and the length of flower lifespan, which importantly affects the probability of pollination [7,8,9,10,11,12]. Given the pivotal role of temperature in plant growth and development, it is critical to understand the factors that determine temperature experienced by plants.

In alpine habitats, interactions involving temperature and plant architecture lead to complex thermal dynamics. Plant temperature is determined by the interplay between air temperature, wind speed, relative humidity, and solar irradiance [13,14], all of which vary substantially according to site-specific factors such as exposure, topography, and substrate [1,15,16,17,18,19]. For example, thermal gradients commonly develop near the ground where the soil absorbs solar radiation and warms the adjacent air through conduction and convection during the day [1]. This warming is intensified by reduced wind speed close to the surface, which limits convective heat exchange and allows heat to accumulate [20,21]. In addition, small-scale topographic features such as depressions, rocks, bare soils, and dark or rough surfaces can generate localized microclimates by trapping air or altering radiation exposure [15]. The temperature experienced by a plant is also shaped by intrinsic factors, such as its growth form, height, and leaf morphology [1,17,22,23], which directly influence biophysical processes such as boundary layer resistance to convective heat transfer and transpiration cooling [24,25,26,27,28]. As a result of all these factors, the temperature experienced by plants can be either warmer or cooler than that of the surrounding air [13,14,24,25,29], a phenomenon known as “thermal decoupling (TD)” (i.e., thermal offset [30,31,32,33,34,35]).

Unlike hot and arid climates, where plants frequently maintain leaf temperatures cooler than the air—sometimes lowered by as much as 17 K [36,37,38]—positive thermal decoupling (plant temperature exceeding air temperature) is a phenomenon that has been extensively documented in alpine plants [1]. This “extra heat phenomenon” is primarily driven by plant dwarfism—a functional syndrome encompassing low stature, compact canopy architecture, and small leaves [1,22,34,39,40,41,42]. The mechanisms underlying positive thermal decoupling are primarily related to the structural capacity of alpine plants to reduce heat loss. Dense, low-stature canopies reduce wind penetration, minimizing convective heat loss, which buffers internal temperatures [6,43,44,45]. Rounded canopy architecture, which is typical among alpine plant growth forms (PGFs), further limits thermal dissipation due to a low surface area-to-volume ratio, which reduces exposure to wind and enhances heat conservation [46,47,48]. Although small leaves may promote greater convective heat loss and thus facilitate foliar cooling due to their thinner boundary layer [37,38,49], they also contribute to dense and compact canopy architectures in alpine species. This structural arrangement reduces internal airflow and enhances thermal buffering, promoting heat retention near the ground [1,14]. Therefore, positive thermal decoupling is particularly strong in low and compact alpine plant growth forms such as cushion plants and rosettes, becoming progressively weaker in taller or more open architectures such as dwarf shrubs [1,17,19].

While heat-trapping plant architecture benefits alpine plants at low temperatures, it can become detrimental during high-temperature events, increasing the risk of heat damage [50,51,52]. Additionally, site characteristics such as wind sheltering, soil water scarcity, and dark substrates can further intensify plant heating, causing plant temperatures to exceed 30 °C and occasionally reach 40 °C [52,53,54,55,56]. This is particularly relevant in alpine ecosystems, which are among the most severely affected by climate change [57,58]. For example, an increase of 0.25 °C per decade in air temperature has been reported in the Chilean Andes [59]. Simultaneously, an upward trend of more than one heat wave event per decade has been reported across the Andes between 20° and 36° S since 1980 [60]. Therefore, heat stress is likely to be common in alpine species, especially in PGFs where the leaves are near the ground or arranged in a dense canopy where the temperature tends to be higher [61,62]. In alpine ecosystems, particularly those influenced by a Mediterranean-type climate, where daytime temperature extremes can be very high, the risk of heat stress may be exceptionally high and pose a significant challenge for plant survival [34,63].

Paradoxically, while positive thermal decoupling in alpine plants is beneficial for facing low temperatures, the possibility of overheating is expected to force alpine plants to invest in heat resistance mechanisms. In other words, alpine plants must withstand both low and high temperatures [1,34,53,64]. Neuner and Buchner (2012) studied 79 alpine species from temperate regions and reported heat resistance ranging from 43° to 64 °C [54]. Similar results were found by León-García and Lasso (2019), who studied 21 species from the tropical Andes [65], and Sumner and Venn (2022) in 11 Australian alpine species [66]. In all these cases, heat resistance varied according to PGF, with grasses and plant rosettes being the most heat-resistant. Although these studies suggest that heat resistance is strongly influenced by plant growth form [67,68,69], to our knowledge, no study has directly evaluated the impact of thermal decoupling as a result of growth form on heat resistance capacity of alpine plants.

Our main goal was to explore the effects of thermal decoupling shaped by environmental factors and plant architectural traits on the heat resistance of common alpine species from the Nevados de Chillán volcanic complex in the Andes of south-central Chile. Our specific goals were (1) to assess the importance of environmental variables on plant temperature; (2) to determine the extent to which plant temperature is decoupled from air temperature at the microclimatic scale; (3) to test the importance of plant architecture traits in explaining thermal decoupling; and (4) to assess the relationship between plant thermal decoupling and heat resistance. We expected to find that thermal decoupling is a key determinant of heat resistance in alpine plant species. In particular, we expected that rosette species—characterized by a growth form that promotes stronger positive thermal decoupling (i.e., canopy temperatures exceeding air temperatures)—would exhibit higher heat resistance. In contrast, dwarf shrub species, which tend to be thermally coupled to ambient conditions due to their more elevated and exposed architectures, were expected to display lower heat resistance.

The alpine flora of the Nevados de Chillán volcanic complex encompasses the primary alpine PGFs, ranging in height from 1 to 90 cm, exhibiting diverse canopy architectures [70]. As the season progresses, moisture in the air and soil decreases, with heat events up to 41 °C becoming increasingly frequent [71]. Projections indicate that summer temperatures could rise by 2–5 °C, and the frequency of heat waves may increase by up to 20 days by the end of this century [59,72]. The increasing frequency and intensity of heat events, compounded by microclimates in alpine environments, pose significant physiological challenges for plants. This raises the crucial question of how alpine plants that are morphologically and physiologically adapted to cold conditions can withstand high temperatures. The findings from this study could provide valuable insights for developing conservation strategies and improving predictive models of alpine plants’ responses to climate change.

## 2. Results

### 2.1. Environmental Variables Affecting Plant Temperature

**Figure 1:**
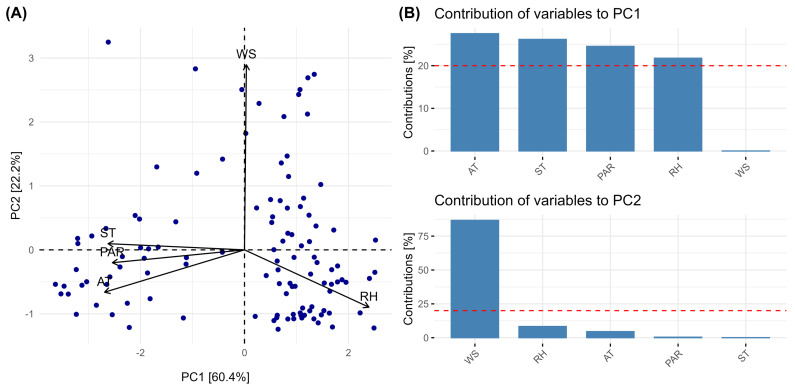
Principal component analysis (PCA) of environmental variables influencing plant temperature. (**A**) Biplot of the first two principal components (PC1 and PC2), derived from five environmental variables: air temperature (AT), photosynthetically active radiation (PAR), relative humidity (RH), soil temperature (ST), and wind speed (WS). The percentage of variance explained by each component is indicated in brackets. (**B**) Bar plots showing the contribution (%) of each environmental variable to the formation of PC1 and PC2. The red dashed line represents the expected average contribution if all variables contributed equally. Variables with contributions above this threshold are considered the most influential in defining each principal component.

High collinearity characterized most of the environmental variables, except for the combinations wind speed (WS)–soil temperature (ST) and WS–photosynthetic active radiation (PAR) (Figure S1). The highest correlations were found for air temperature AT–ST (0.68), PAR–AT (0.64), and PAR–relative humidity (RH) (−0.64). The lowest correlations were found for WS–AT (−0.25) and WS–RH (−0.22) (Figure S1). Due to high collinearity between the explanatory variables, principal component analysis (PCA) was performed. We used the principal factors PC1 and PC2 to summarize the environmental data to model plant temperature. PC1 and PC2 accounted for about 60% and 22% of the variability of the environmental variables used, respectively (Figure 1A).

PC1 was negatively related to AT, ST, and PAR. To a lesser extent, it was positively correlated to RH. PC2 was negatively correlated to WS and positively correlated to RH (Figure 1B). Hence, PC1 reflected the input of solar energy and heat transfer, whilst PC2 reflected the heat loss through the WS.

The generalized additive model (GAM) explained a substantial proportion of the variance in plant temperature (adjusted R^2^ = 0.80, *p* < 0.0001; Table 1).

The model identified a significant non-linear relationship between PC1 and plant temperature (degrees of freedom = 6.89, F = 57.32, *p* < 0.0001), indicating that PC1 captured most of the variation explained by the model. In contrast, the effect of PC2 was not statistically significant (*p* = 0.11). Based on the correlations between environmental variables and PC1 (negatively related to air and soil temperature and PAR, positively related to relative humidity), the results suggest that higher solar radiation and warmer air and soil temperatures increase plant temperature, whereas higher relative humidity reduces it.

### 2.2. Plant Thermal Decoupling

**Figure 2:**
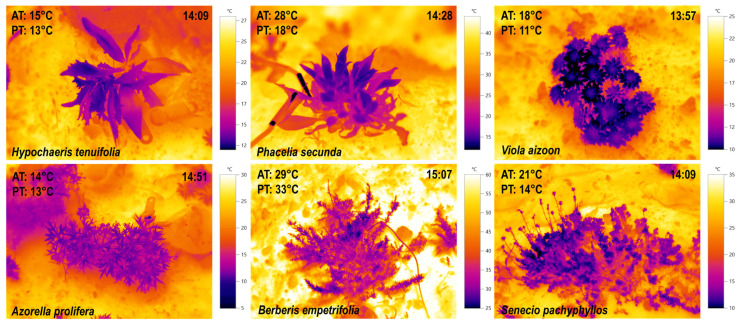
Infrared (IR) images of the six alpine study species. Images were taken between 14:00 and 15:00 (P2) on a north-facing slope during three sampling days (7, 8 March, and 12 April 2023). Rosette species are shown in the upper row, and dwarf shrubs in the lower row. Air temperature (AT, °C) and plant temperature (PT, °C) at the time of capture are shown in the upper left corner of each image, while the exact capture time is displayed in the upper right. Species names are indicated in the lower left corner of each panel.

**Figure 3:**
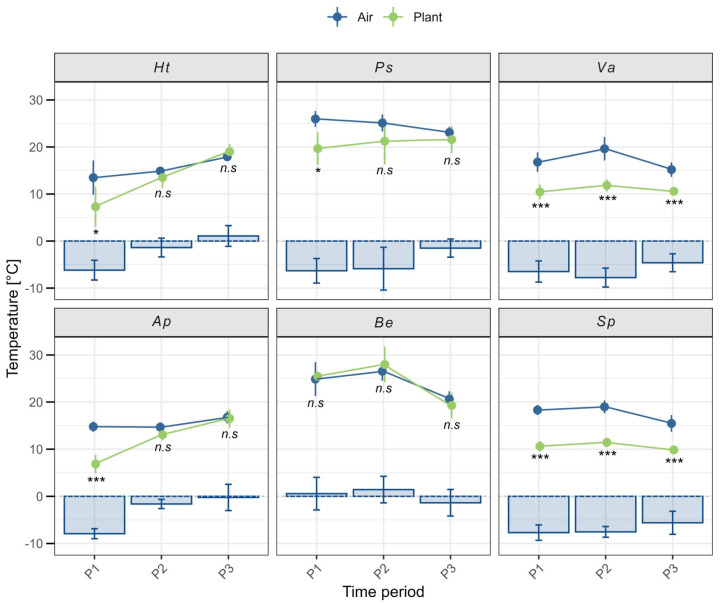
Air (blue) and plant (green) temperatures measured across three time periods: P1 (10:00–12:00), P2 (13:00–15:00), and P3 (16:00–18:00). For *Berberis empetrifolia*, P1 corresponds to 12:00–13:00. Species abbreviations: rosettes—Ht (*Hypochaeris tenuifolia*), Ps (*Phacelia secunda*), Va (*Viola aizoon*); dwarf shrubs—Ap (*Azorella prolifera*), Be (*Berberis empetrifolia*), Sp (*Senecio pachyphyllos*). Circles represent mean temperatures; whiskers are the standard deviation (SD). Blue bars represent mean thermal decoupling (TD = PT *−* AT), shown with SD. Statistical significance of TD is indicated as follows: *** *p* < 0.001, * *p* < 0.05, and *n.s.* = not significant.

Negative thermal decoupling (TD = PT-AT) was observed in five of the six species studied (Figure 2), with plant temperature (PT) averaging 6.7 K lower than air temperature (AT) (blue bars in Figure 3).

A strong sampling-day effect on TD was detected, with species measured on the same day exhibiting similar TD patterns (Figure 3). In *Hypochaeris tenuifolia* and *Azorella prolifera*, TD occurred only in the morning (P1), with PT averaging 6.2 ± 2.1 °C (t_10_ = 2.71, *p* = 0.02) and 7.9 ± 1.1 °C (t_10_ = 8.79, *p* < 0.0001) lower than AT, respectively (Figure 3). A similar pattern was observed in *Phacelia secunda*, where PT was, on average, 6.3 ± 2.6 °C lower than AT at P1 (U = 4, *p* = 0.02). Although TD in *Berberis empetrifolia* was measured the same day as in *P. secunda*, PT and AT in the former species remained coupled throughout the day (P1–P3) (Figure 3; P1: t_6_ = −0.3; P2: t_10_ = −0.8; P3: t_10_ = 1.1, *p* > 0.05). The apparent TD difference at P1 between *B. empetrifolia* and *P. secunda* may have resulted from the time shift of P1 (12:00–13:00) in *B. empetrifolia*, which aligned more closely with P2 in *P. secunda*.

In *Senecio pachyphyllos* and *Viola aizoon*, TD occurred throughout the day, with PT being consistently lower than AT across all periods (Figure 3). In *S. pachyphyllos*, PT was 6.9 ± 2 °C lower than AT (P1: t_12_ = 13.1; P2: t_12_ = 11.2; P3: t_12_ = 7.5, *p* < 0.0001). Similarly, in *V. aizoon*, PT was 6.2 ± 2.4 °C lower than AT (P1: t_12_ = 6.5; P2: t_12_ = 7.3; P3: t_12_ = 7.2, *p* < 0.0001). Thermal decoupling (TD), calculated as daily mean values per species across the three measurement periods, did not differ significantly between growth forms. Rosette species had a mean TD of −4.6 K (±2.3), while dwarf shrubs had a mean TD of −3.8 K (±3.2) (t_34_ = −0.86, *p* = 0.396).

### 2.3. Thermal Decoupling (TD) and Architecture Plant Traits

The relationship between plant architectural traits and TD was analyzed by averaging TD per species during daytime hours (P1–P3). A linear model (LM) was fitted with plant height (PH), circularity index (CI), and porosity index (PI) as predictors, and sampling date as a fixed cofactor. The overall model was significant (F_5, 21_ = 11.55, *p* < 0.0001) and explained 67% of the variability in TD (Table 2).

PH had a significant effect, accounting for 24% of the variance in TD (F_1,21_ = 18.80, *p* = 0.0003), while CI explained 19.2% (F_1,21_ = 15.12, *p* = 0.0008). These results suggest that smaller, more rounded plants exhibited more negative thermal decoupling (TD) values than taller, less rounded individuals, indicating stronger cooling relative to air temperature. The sampling date also contributed significantly, explaining 25.2% of the variance (F_1,21_ = 19.82, *p* = 0.0002), reinforcing the pattern shown in Figure 3.

### 2.4. Relationship Between Thermal Decoupling (TD) and Heat Resistance (LT_50_)

**Figure 4:**
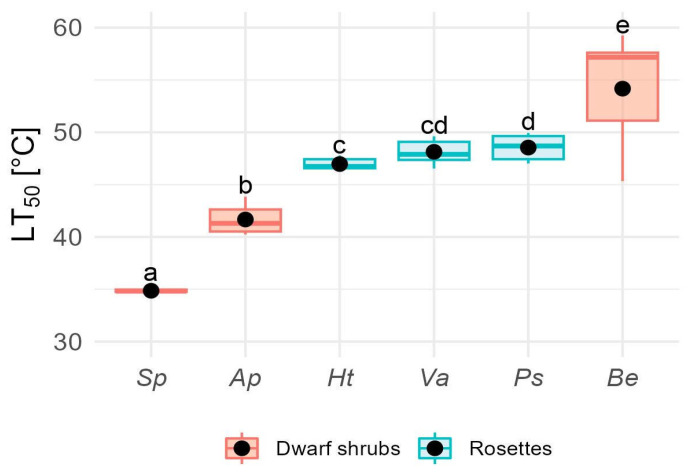
Leaf heat resistance (LT_50_, °C) in six alpine species. LT_50_, defined as the lethal temperature at which 50% of tissue damage occurs, was estimated for rosette species Ht (*Hypochaeris tenuifolia*), Ps (*Phacelia secunda*), Va (*Viola aizoon*) and dwarf shrub species Ap (*Azorella prolifera*), Be (*Berberis empetrifolia*), Sp (*Senecio pachyphyllos*). Box plots show the median (line inside the box), interquartile range (IQR; box limits), and whiskers extending to 1.5 × IQR. Circles indicate mean values. Different superscript letters denote statistically significant differences in LT_50_ among species (*p* < 0.05).

Mean LT_50_ varied significantly among species (F_5,40_ = 13.8, *p* < 0.0001), ranging from 35.2 ± 0.3 °C in *S. pachyphyllos* to 54.2 ± 1.9 °C in *B. empetrifolia*. Notable differences in LT_50_ were observed among shrub species, which exhibited a range of 19 K (Figure 4).

In contrast, there was little variation in heat resistance among rosette species (Figure 4). Interestingly, the LT_50_ for rosette species was 4 K higher than that of dwarf shrubs (H_1,40_ = 4.68, *p* = 0.03), with a mean LT_50_ of 47.9 ± 1.2 °C and 43.9 ± 8.7 °C for rosette plants and dwarf shrubs, respectively.

**Figure 5:**
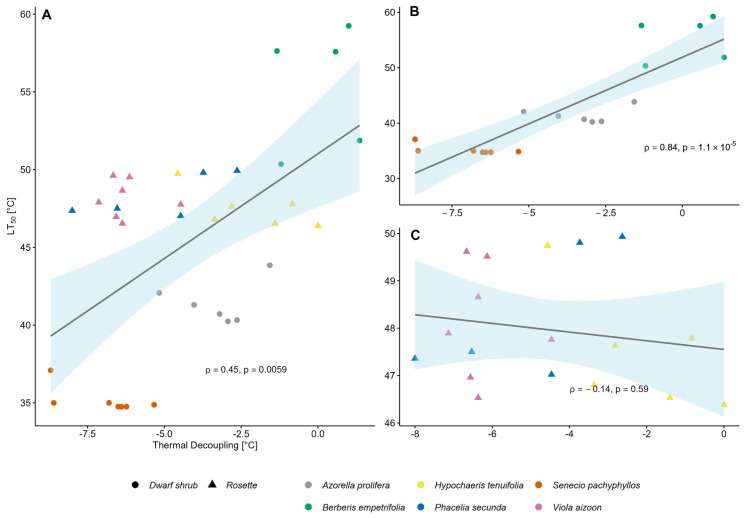
Relationship between thermal decoupling (TD) and heat resistance (LT_50_) across alpine species. (**A**) Correlation between TD and LT_50_ for all species combined; (**B**) rosette species (triangles); and (**C**) dwarf shrub species (circles) analyzed separately. Thermal decoupling (TD) corresponds to the mean difference between plant and air temperature, averaged per individual across three daytime measurement periods (between 10:00–18:00). LT_50_ refers to the lethal temperature at which 50% tissue damage occurred. Different colors represent different species. Spearman’s rank correlation coefficient (ρ) and corresponding *p*-value are shown at the bottom of each plot.

A positive correlation was found between plant thermal decoupling and heat resistance (Figure 5A; ρ = 0.45; *p* = 0.006). Hence, those individuals with negative TD values (i.e., PT < AT) had a lower LT_50_ than those with higher TD values (i.e., PT similar to AT; Figure 5A).

Whereas most dwarf shrubs (denoted by circles) aligned with this positive correlation (Figure 5B; ρ= 0.84; *p* < 0.0001), most of the rosette plants (represented by triangles) did not conform to this pattern (Figure 5C; ρ = −0.14; *p* = 0.6).

## 3. Discussion

We studied the ability of plants to decouple from environmental temperatures, using microclimatic and plant trait characterization combined with infrared thermal images. Our findings provide new insights into plant thermal behavior and heat resistance, highlighting the complex interactions between microclimatic conditions and plant traits in alpine environments. Despite the heat-trapping design traditionally associated with alpine species, most of the plants studied in this Mediterranean-type climate alpine area were, on average, 7 °C cooler than the ambient temperature. Levels of negative thermal decoupling varied throughout the day and between species. Interestingly, species with stronger negative thermal decoupling tended to be less heat-resistant, although this pattern was evident only in shrub species. Nevertheless, most species exhibited high levels of heat resistance, suggesting that heat stress imposes a strong adaptive pressure in this alpine region. Although this study was limited to six species, our results challenge the assumption that alpine plants are consistently positively decoupled during the day and underscore the importance of considering microclimatic influences when assessing plant thermal responses. At the same time, our results contribute to a better understanding of how alpine plants cope with heat stress, an aspect often overlooked in alpine ecosystems due to the predominant focus on cold temperatures.

As expected, plant temperatures were the results of the combined effects of solar irradiance, microclimate air temperatures, and relative humidity. This pattern aligns with the energy balance theory [46], which posits that solar radiation absorption primarily drives the energy balance in leaves [5,17,35,73]. In addition, plants gain heat through convection from the surrounding air, infrared radiation from the soil, and heat conduction between the soil and plant parts in contact with it [13,74,75,76]. On the other hand, the observed decrease in plant temperature with increasing relative humidity may be attributable to the higher water vapor content in the air, increasing the probability of cloud formation [77]. Water vapor and clouds absorb and scatter solar radiation, reducing direct solar radiation reaching the plants. This relationship is further supported by a negative correlation between photosynthetic active radiation (PAR) and relative humidity (RH) identified in this study (Figure S1). Although most of the variance (77%) in plant temperature was explained by air/soil temperature and PAR, plant traits such as height and canopy architecture significantly affected the temperature experienced by plants and contributed to their ability to decouple from environmental temperatures.

The finding that species underwent negative thermal decoupling is contrary to what might have been expected in alpine growth forms, particularly for dwarf shrubs and rosettes, which have been described as heat traps, i.e., with positive thermal decoupling, PT > AT [1,13,14,22,34,55]. Interestingly, negative thermal decoupling has been described for plants inhabiting hot and arid climates where heat stress is prevalent [36,37,38,78]. This has been explained by latent heat loss through transpiration provoking a cooling effect in these species [74,79]. Although stomatal conductance was not assessed in this study, vapor pressure deficits (VPD) registered during the thermal decoupling field measurements (Figure S2) indicated a moderate to high potential for stomatal transpiration rates [80,81]. Under moderate VPD levels (<1.5 kPa), thermal decoupling was negatively correlated to VPD (Figure S3; R^2^ = −0.48; *p* < 0.0001); therefore, plant temperature decreased when VPD increased. However, at higher levels of VPD (2.3 ± 0.5 kPa), such as those observed during TD measurements in *Berberis empetrifolia* and *Phacelia secunda*, plants’ temperatures were generally coupled with air temperature, probably due to stomatal closure to reduce water loss and enhance the efficiency of water use [82]. This suggests that thermal decoupling capacity depends not only on environmental conditions but also on the plant’s water status and soil moisture availability [83]. Exceptions to this trend were noted for *Azorella prolifera* and *Hypochaeris tenuifolia*, where negative TD did not solely respond to VPD. These varying responses among species might be related to specific traits influencing transpiration sensitivity to changes in VPD [84,85,86,87], such as anatomical features like variations in stomatal size, density, and guard cell activity [88,89,90]. Overall, the observed thermal decoupling capacity appears to have been predominantly influenced by environmental factors. This conclusion is supported by the pronounced sampling-day effect on TD, with species measured on the same day exhibiting similar TD patterns (Figure 3). Given this observation, the thermal decoupling patterns reported here should be interpreted as a temporal snapshot of environmental conditions during the measurement period. Whether these patterns remain consistent over broader temporal scales from daily and seasonal fluctuations to interannual climate variability remains an open question that warrants further investigation.

Our findings on negative thermal decoupling shed light on a critical methodological issue. Studies on thermal decoupling have mostly assessed this phenomenon using weather stations located two meters above ground, with a few notable exceptions in tropical [91,92] and temperate alpine species [93]. These studies have concluded that alpine plants function as heat traps [33,94,95,96,97]. However, the use of weather station data is debatable given that the short stature of alpine plants exposes them to a microclimate that can differ significantly from the conditions recorded at standard meteorological heights [17,61,68,98]. In this regard, comparing our results for plant temperature with those for air temperature at the macroscale, PT averaged 2.3 K higher than AT (Figure S4). This evidence suggests that the direction of thermal decoupling—positive or negative—depends on where the air temperature is measured, highlighting the need for careful interpretation of results and adequate representation of the real air temperature around plants. Although our TD measurements were taken at specific times of day (and would have benefited from more extensive sampling across the diel cycle and increased replication to improve statistical power), the results clearly suggest that alpine plants do not consistently act as heat traps. On the contrary, we found that TD measurements based on air temperatures at the height of the plant can lead to conclusions that suggest a cooling effect in alpine plants. These findings underscore the urgent need to standardize methodologies for assessing thermal decoupling, particularly regarding the height of air temperature measurements. Such standardization is essential in order to ensure comparability across studies and to enhance our understanding of plants’ thermal responses. Precise, plant-level measurements that accurately capture the microclimatic conditions experienced by plants are essential to uncover the adaptive strategies of plants in extreme environments.

In addition to the influence of environmental conditions on TD, as evidenced by the pronounced sampling-day effect on thermal decoupling, the extent of thermal decoupling is also modulated by plants’ architectural traits, with smaller, more rounded growth forms exhibiting the greatest negative TD. Shorter species like *Viola aizoon* and *P. secunda* exhibited stronger negative TD than taller plants like *B. empetrifolia*, which suggests that shorter plants may be more influenced by soil temperature. Stronger negative thermal decoupling in shorter species may help maintain leaf temperatures within the optimal range of carbon assimilation and growth temperature. This is particularly relevant since their closer thermal coupling with near-ground conditions can expose them to rapid temperature increases in high irradiance conditions. In fact, soil temperatures close to 40 °C have been recorded at this study site [99]. Conversely, *B. empetrifolia*, the tallest shrub, appeared less influenced by soil temperature and its temperature was more correlated with air temperature, and this was the only species where wind speed correlated with plant temperature (R^2^ = 0.41, *p* = 0.005). Furthermore, the canopy as expressed by the circularity index (CI) explained 16.3% of the variance in thermal decoupling. Interestingly, species at both extremes of plant height coincided with variations in circularity. *V. aizoon* and *P. secunda* ranked as the most rounded, while *B. empetrifolia* was the least rounded. The stronger negative TD observed in rounded plants may be associated with their compact leaf arrangement and horizontal leaf orientation. These traits could provoke leaf heating by enhancing absorption of solar radiation [93,100,101], thereby driving higher transpiration rates and a stronger negative TD. These findings underscore that while environmental factors affect plant temperature, whole plant/leaf traits can mitigate or amplify these effects [24].

We initially expected alpine plants to overheat due to positive TD, requiring them to maintain high heat resistance. Contrary to this expectation, most species displayed relatively high heat resistance (mean LT_50_ of 46 °C) while exhibiting negative TD. In general, the LT_50_ values observed in our study aligned well with those reported for other alpine plants [54,56,65,66,102]. Heat resistance was particularly variable among dwarf shrubs, ranging from 35 to 54 °C. The low LT_50_ of 35 °C in *Senecio pachyphyllos* was notably below the mean LT_50_ of alpine shrub species, reported at 47 °C [102]. In contrast, *B. empetrifolia* exhibited an exceptionally high heat resistance of 54 °C compared with other shrub species [102]. In dwarf shrubs, at least two factors may explain these differences in LT_50_. First, TD partially explained the variation in LT_50._ The strong negative TD observed in *S. pachyphyllos* effectively prevents its leaves from being exposed to extreme high temperatures, resulting in a lower LT_50_. Conversely, *B. empetrifolia*, where the plant temperature closely tracked the air temperature, had the highest LT_50_. This pattern suggests that the degree of TD partially explains the variation in LT_50_ among shrub species, with more pronounced negative TD providing protection against heat stress. Second, leaf morphological traits are likely to underpin the LT_50_ values observed in shrubs. *A. prolifera* and *B. empetrifolia* exhibit typical sclerophyllous anatomy, characterized by rigid, dense tissues and low water content [103]. These leaf structural traits may enhance heat resistance by minimizing the risk of cellular collapse under thermal stress by maintaining tissue stability at high temperatures [67,104]. In contrast, *S. pachyphyllos* possesses relatively thick but soft leaves with higher apparent water content [105], suggesting a more water-dependent, heat-avoidant strategy. In this case, increased water content may buffer short-term temperature rises through thermal inertia and facilitate a greater capacity of negative TD. Although we did not directly measure leaf morphological traits, the observed differences suggest that leaf morphoanatomy could influence heat tolerance across species, warranting further investigation.

Rosette species, by contrast, showed consistently high LT_50_ values, averaging 48 °C, comparable to those found in erect (47.8 °C) and acaulescent (49.5 °C) rosette species from tropical and temperate alpine habitats [102]. Unlike shrubs, no correlation was found between TD and LT_50_ in rosette species. The lack of a consistent correlation between thermal decoupling (TD) and LT_50_ suggests that cooling through transpiration may not be a targeted heat resistance mechanism but a consequence of water loss associated with stomatal opening required for photosynthesis [83]. In this context, leaf traits such as thickness, width, and surface area probably play a more prominent role in buffering leaves against extreme temperatures by affecting heat storage capacity and boundary layer properties [23,28,106]. Rosette plants, which are exposed to more intense and prolonged thermal extremes than taller species [62,107], probably rely on these morphological traits alongside biochemical mechanisms of heat tolerance (e.g., heat shock proteins, osmotic adjustment, antioxidant production) to withstand such thermal conditions. Although negative thermal decoupling temporarily reduces leaf temperatures, it may not be sufficient for survival under severe heat stress. Nonetheless, further investigation is required to clarify the exact contribution of negative TD to thermal resilience.

Rosette species and *B. empetrifolia* have a wide safety margin to cope with heat events. Their mean heat resistance is around 50 °C (Figure 4), which is 16 °C above the average heat intensity recorded during the growing season at the study site, i.e., 33.5 °C [71]. In contrast, the shrub species *S. pachyphyllos* (LT_50_ = 35 °C) and *A. prolifera* (LT_50_ = 42 °C) have narrow safety margins, making them more vulnerable to extreme heat. Given that air temperature exceeded 30 °C for 73 days during the growing season in our study site, with peaks reaching 41 °C at the microclimate scale [71], the cooling effect from TD is critical for these species to withstand heat events. However, relying on TD as a “heat avoidance mechanism” poses risks, especially as future projections indicate that this alpine zone will become hotter and drier [59,108], making it harder for plants to cool down via transpiration [109,110]. Increased temperatures and water shortage could trigger stomatal closure to prevent excessive water loss [82,111], resulting in negative carbon balances and carbohydrate depletion [112]. Furthermore, a combination of water shortages and high evaporative demand might lead to cavitation and plant mortality [113,114]. Thus, maintaining high levels of heat resistance and taking advantage of thermal decoupling as a “heat avoidance mechanism” may be essential for plant survival in the face of climate change in this and other alpine systems experiencing heat and drought events.

In conclusion, this study, conducted in an alpine ecosystem influenced by a Mediterranean-type climate, enhances our understanding of plant survival strategies in extreme environments. It highlights that alpine plants’ responses to climate change are likely to vary considerably depending on the specific alpine climate, species identity, and the unique microclimatic conditions they experience. Our findings demonstrate that multiple environmental factors and whole-plant traits influence plants’ temperature and their ability to cope with heat events. A particularly novel and underexplored aspect is the paradoxical finding that many species still exhibited high heat resistance despite the widespread observation of negative thermal decoupling. This highlights the complexity of plants’ thermal regulation and suggests that multiple, complementary strategies operate simultaneously to enhance tolerance to high temperatures. Given the strong dependence of plant temperature on water availability, future research should address seasonal variations in thermal decoupling and heat resistance and directly investigate the relationship between transpiration and thermal decoupling to elucidate the underlying mechanistic processes. This will be particularly relevant in alpine regions prone to summer drought, such as the Chilean Andes, Snowy Mountains (Australia), Rocky Mountains (USA), Sierra de Guadarrama (Spain), and East Pamirs (Central Asia). Understanding water-mediated thermal tolerance holds broad ecological relevance in these systems, where climate change has been shown to impact plant diversity more severely than in temperate alpine environments [115]. Moreover, a more comprehensive temporal sampling spanning different times of day, stages of the growing season, and slope aspects is needed to characterize thermal decoupling patterns under diverse climatic conditions. Finally, it remains essential to examine whether the thermal behavior observed in these species also contributes to their ability to withstand freezing temperatures, a key environmental filter in alpine ecosystems.

## 4. Materials and Methods

### 4.1. Study Site and Plant Species

This research was conducted in the Aguas Calientes Valley, located within the Nevados de Chillán Volcano complex, approximately 80 km east of Chillán (Región del Ñuble, Chile). This valley lies in the southern part of the mediterranean-type climate zone of central Chile, transitioning into the southern temperate forest region [116]. The area is characterized by both past and ongoing volcanic activity, resulting in complex topography, geology, and a variety of substrate types [117,118]. These climatic and geomorphological conditions contribute to exceptional plant diversity and endemism [119]. The vegetation consists of dwarf shrubs, rosette herbs, grasses, and geophytes [120,121]. Our study site was located just above the treeline on a north-facing slope at an elevation ranging from 1941 to 2003 m above sea level (36°54′22.46″ S, 71°24′10.30″ W). The vegetation was dominated by dwarf shrubs (e.g., *Azorella prolifera, Berberis empetrifolia, Discaria chacaye*), rosette perennial herbs (e.g., *Viola cotyledon, Perezia pilifera, Phacelia secunda*), and grasses (e.g., *Koeleria barbinodis, Poa denudata*) [120,121,122].

The nearest meteorological station (Termas de Chillán, CN360042; 1708 m; https://climatologia.meteochile.gob.cl/, accessed on 12 March 2023) reported average annual precipitation ranging from 615 to 1012 mm over the past decade, most of which fell during winter as snow. During the growing season, mean minimum and maximum air temperatures were 0.8 °C and 23 °C, with recorded extremes ranging from −6.6 °C to 28.5 °C. At the microclimatic scale, particularly on the north-facing slope where the study site was located, average minimum and maximum air temperatures reached 2.4 °C and 26 °C, with absolute extremes ranging from −9.9 °C to 41.3 °C [71]. At 15 cm above ground level, the mean temperature of heat events was 34 °C, and 49% of days during the growing season experienced temperatures above 30 °C for at least three consecutive hours. Additionally, soil moisture decreased 2.5-fold from October to March, exposing plants to progressive water limitation [71]. North-facing slopes in this region experience higher maximum temperatures and more frequent and intense heat events than their south-facing counterparts [71]. This selection minimized variation associated with the slope aspect and ensured consistent microclimatic exposure across sampling sites.

**Figure 6:**
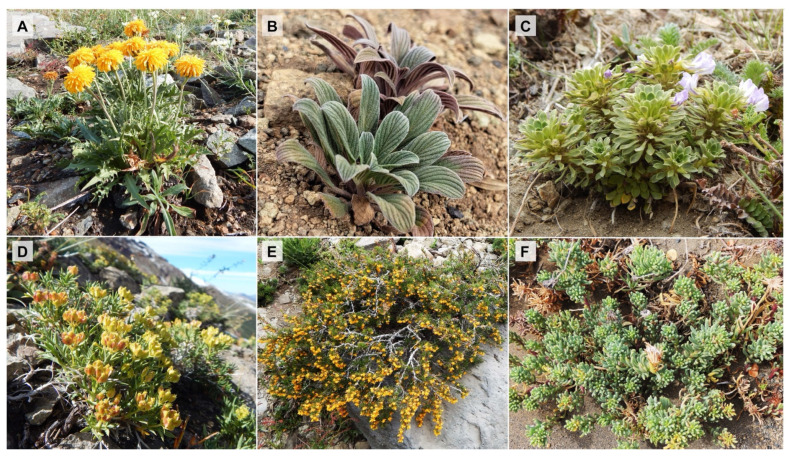
Plant species studied in the alpine belt of the Nevados de Chillán Volcano complex. In the upper section, the rosette species are (**A**) *Hypochaeris tenuifolia*, (**B**) *Phacelia secunda*, and (**C**) *Viola aizoon*. In the lower section, the dwarf shrub species are (**D**) *Azorella prolifera*, (**E**) *Berberis empetrifolia*, and (**F**) *Senecio pachyphyllos*.

Six native species of two plant growth forms (PGFs), i.e., plant rosettes and dwarf shrubs, were studied (Figure 6). These species were chosen because of their high relative abundance on the north-facing slope [122] and their individual structures, which enabled accurate measurement of whole-plant temperature using infrared thermography. To avoid temperature and canopy overlap, species forming spatial associations were excluded. The selected species also represent the dominant alpine growth forms (rosettes and dwarf shrubs), enabling us to examine contrasting thermal and morphological strategies effectively.

The rosette plant species were *Hypochaeris tenuifolia* (Hook. and Arn.) Griseb (*Asteraceae*), *Phacelia secunda* J.F. Gmel. (*Boraginaceae*), and *Viola aizoon* Reiche (*Violaceae*). *H. tenuifolia* is a rhizomatous perennial herb with rosette leaves, 3–21 cm tall, with leaves that are glabrous, entire to pinnate, 5–10 cm in length [123]. *P. secunda* is a perennial herb with a basal rosette 6–7 cm tall, with an upright rhizome; its basal leaves are long petiolate, hairy on both sides, and up to 8 cm long [124]. *V. aizoon* is a perennial herb with succulent, glabrous, semi-spreading leaves that form 4 cm tall rosettes. The leaves are stiff and membranous, with short petioles [125]. The dwarf shrub species were *Azorella prolifera* (Cav.) G.M. Plunkett and A.N. Nicolas (*Apiaceae*), *Berberis empetrifolia* Lam. (*Berberidaceae*), and *Senecio pachyphyllos* J. Remy (*Asteraceae*). *A. prolifera* is a dwarf shrub <100 cm tall that forms lax cushions. Its leaves are 1–5 cm in length, numerous, alternate, and petiolate [126]. *B. empetrifolia* is a small-thorny, mostly prostrate shrub <50 cm tall with angular, glabrous, spiny branches and scaly bark in mature plant stages [127]. *S. pachyphyllos* is a many-branched dwarf shrub, 15–20 cm tall, with creeping rooting stems and leaves crowded at the base of the plant [128].

### 4.2. Environmental Variables, Plant Temperature, and Thermal Decoupling

A thermal imaging camera (Testo 885, Testo INC, Lenzkirch, Germany), featuring a thermal sensitivity (NETD) of <30 mK at 30 °C and a temperature accuracy of ±2 °C, was used to measure the temperature of six individuals per species. Infrared (IR) images were taken three times during daylight hours (P1: 10–12 h, P2: 13–15 h, and P3: 16–18 h) on 7 March (*B. empetrifolia* and *P. secunda*), 8 March (*A. prolifera* and *H. tenuifolia*), and 12 April (*S. pachyphyllos* and *V. aizoon*) 2023. Infrared images were captured at the closest focal plane to ensure a comprehensive representation of the entire plant. The plant emissivity in the infrared camera was set to 0.98 [129,130,131]. The distance and angle of capture were adjusted according to species size, with distances less than 10 cm for rosettes and up to 50 cm for dwarf shrubs. The transmissivity of the atmosphere was not set during measurements because the camera was positioned close to the plants. As a result, this parameter is expected to have had a minimal effect on the thermography data [131]. Infrared images were taken during clear or partly cloudy days (mean air temperature: 19.3 ± 4.9 °C; mean relative humidity: 37 ± 13.5%). For precise details of the timing and environmental conditions during all thermal measurements, see Table S1.

Concomitantly with the plant temperature measurements, three environmental variables were measured at the level of the target plant (microclimate) using a Kestrel 3000 Pocket Weather meter (Nielsen-Kellerman Co., Ltd., Boothwyn, PA, USA): air temperature (AT, °C; accuracy ± 0.5 °C), relative humidity (RH, %; accuracy ± 3%), and wind speed (WS, km h^−1^; accuracy ± 3%). Because the species varied in height, these variables were measured at different distances from the ground. Simultaneously, photosynthetic active radiation (PAR, µmol m^−2^ s^−1^) was measured using a LI-250Q Light Meter (Li-Cor, Lincoln, NE, USA). The light meter was aligned parallel with the leaf surfaces, ensuring that the orientation matched that of the leaves, particularly those located at the upper canopy level in dwarf shrub species. This alignment was critical for capturing the accurate light conditions experienced by the photosynthetic tissues. All measurements were executed within a 10 s interval per plant to minimize any effect of solar radiation on air temperature measurements.

Six visually healthy individuals per species were selected to ensure adequate replication for thermographic imaging and concurrent environmental measurements under standardized conditions. Individuals were spaced 50–100 cm apart to ensure they represented genetically and physically distinct plants, while still sharing similar microhabitat conditions (e.g., substrate). Thermal and environmental data were collected from two species per session, with 16 individuals measured over a two-hour period (approximately 7 min per replicate), resulting in 36 replicates per day. This design ensured consistent environmental and habitat sampling conditions across all replicates and species.

To determine plant temperature (PT, °C), IR image analysis was performed using IRSoft version 5.0 software (Testo INC, Lenzkirch, Germany). The plant area, including all vegetative parts, was contoured manually to obtain plant surface temperature. Soil temperature (ST, °C) was estimated from the free-plant soil area of the same infrared images used to estimate PT. Thermal decoupling (TD, °C) was calculated for each plant by determining the difference between plant temperature (PT) and air temperature (AT).

### 4.3. Plant Architecture Traits

Plant height, circularity, and porosity were measured for each species (i.e., six species × 4–5 replicates). Plant height (PH, cm) was measured as the shortest distance between the upper boundary of leaves (excluding inflorescences) and the ground level using a tape measure [132]. The porosity index (PI) and circularity index (CI) were estimated through images analyzed with ImageJ software v1.53t (Wayne Rasband, NIH, Bethesda, MD, USA; Figure S1). PI is a modification of the gap fraction proposed by Pérez-Harguindeguy et al. (2013) [132]. Due to the prostrate nature of alpine plants, photos were taken above the canopy rather than below it, as was also carried out for the gap fraction.

PI was estimated as follows:

PI = 1 − ((Σ Plant areas)/(height × width))(1)

PI corresponds to the fraction of the plant canopy occupied by air space, where a value of 0 indicates no free spaces in the plant canopy and hence, a dense canopy. As the value approaches 1, free spaces increase in the canopy. Dense canopies, from a functional perspective, reduce light and wind penetration and can provide insulation against extreme temperature fluctuations, creating a more stable microclimate inside the canopy [6,43,44,45].

The circularity index (CI) was estimated as follows:

CI = 4π (Plant area)/(Plant perimeter^2^) (2)

A CI value equal to 1 indicates a perfect circle, while values approaching 0 indicate an increasingly elongated canopy. Rounded shapes have a smaller surface area relative to their volume than other sharp forms [48], which means less surface exposure to the surrounding air, reducing heat loss by convection [46]. In addition, a rounded shape reduces direct radiation exchange by decreasing the plant’s exposed surface area to sunlight, thus minimizing heat absorption [47].

### 4.4. Heat Resistance Assessment

Six fully expanded and healthy leaves were collected from each of the seven haphazardly selected individuals per species to determine heat resistance. For the dwarf shrubs, small branches were collected from the outer canopy, and leaves were detached prior to determinations. Each leaf/branch was immediately covered with a moist paper towel, placed inside a sealed plastic bag, and stored in a cooler to minimize changes in water status. Samples were transferred to a domestic refrigerator within 90 min after collection and kept at approximately 4 °C in until processing. Heat resistance measurements were conducted in field laboratory within 40 h of collection. Before the assays, all samples were visually inspected to confirm tissue integrity and the absence of dehydration.

A cryostat (F34-ME, Julabo Labortechnik GmbH, Seelbach, Germany) with a thermostable solution (Polycool HC-50, Polyscience, Niles, IL, USA) was used to expose leaves to five independent heating treatments: 25, 35, 45, 55, and 60 °C. The five temperature treatments were selected to encompass the full range of physiological heat responses, from mild stress to severe tissue damage [52,53,65,102,133]. Specifically, 25 °C was included as a near-optimal reference temperature, while 35 °C and 45 °C represented sublethal stress levels where early heat damage and reductions in photosynthetic efficiency (e.g., Fv/Fm) are often observed. Temperatures of 55 °C and 60 °C reflected critical thresholds that induced irreversible membrane destabilization and cellular damage [134].

Each treatment used six leaves from seven individuals per species, including the control. Leaves were cut near the base of the petiole, placed inside hermetically sealed plastic bags, and introduced into the cryostat. Heat simulations began at 15 °C, with a heating rate of 10 K h^−1^, and each target temperature was reached within 1–4.5 h. After reaching the target temperature, the samples were maintained for 2 h at that temperature, then returned to 15 °C at the same heating rate of 10 K h^−1^ within the cryostat. Previous studies have used heating rates ranging from 2 K h^−1^ [135] to 60 K h^−1^ [56], indicating a lack of consensus on the most appropriate rate for assessing plant heat resistance. We selected a rate of 10 K h^−1^, a common heating rate in the study area [71,99]. The control leaves were stored in a domestic refrigerator at approximately 4 °C for 17 h, matching the duration of the longest heating treatment (60 °C), ensuring adequate time for leaf damage to be expressed. Even after this period, the Fv/Fm values measuring the photosynthetic efficiency remained above 0.8 in the control samples, indicating that the photosynthetic tissues remained active and undamaged [136].

Temperature-induced leaf damage was assessed 6 h after the completion of each heating treatment. During this period, leaves were kept in darkness inside a domestic refrigerator at approximately 4 °C to prevent further physiological activity and allow dark adaptation. A chlorophyll fluorimeter (Plant Efficiency Analyzer, Hansatech, Reutlingen, Germany) was used to measure the ratio of variable to maximum fluorescence (Fv/Fm) in dark-adapted leaves [136]. Damage was quantified as the percentage of photoinactivation (PhI), calculated using Equation (3):

PhI = (1 − FhT/Fmax) × 100(3)

where FhT is the Fv/Fm of the sample exposed to the heating temperature T, and Fmax is the Fv/Fm value of the control sample. Including Fmax in the equation allows the incorporation of any effect of manipulation and storage time, allowing heat-induced damage to be isolated and directly quantified. Based on the response curves of Fv/Fm to heating treatments (Figure S5), the lethal temperature at which 50% damage occurred (LT_50_) was estimated by linear interpolation between the highest temperature at which the photosynthetic index (PhI) remained below 50% and the lowest temperature at which it exceeded 50%, following an approach modified from previous work [137,138]. LT_50_ was estimated for each of the seven individuals per species, and the mean and variability were then calculated at the species level.

### 4.5. Statistical Analysis

All statistical analyses were performed in R (version 4.2.3, 2023-03-15 ucrt). To evaluate the influence of environmental variables on plant temperature, we performed principal component analysis (PCA) using the prcomp function in R [139], to reduce dimensionality and summarize the variations in air and soil temperature, photosynthetically active radiation (PAR), relative humidity (RH), and wind speed. The first two principal components (PC1 and PC2) were used as predictors in a generalized additive model (GAM), fitted with the gam function from the mgcv package [140], using a Gaussian distribution and identity link function. PC1 and PC2 were included as smooth terms for potential non-linear effects. Model assumptions were evaluated using both graphical and statistical approaches. The normality of residuals was assessed with the Shapiro–Wilk test (W = 0.978, *p* = 0.053), and homoscedasticity was evaluated using the studentized Breusch–Pagan test (*p* = 0.945) and the non-constant variance test (*p* = 0.885). The basis dimension (k) for each smoothing term was tested using the gam.check function. The k-index values (PC1: 1.13, *p* = 0.86; PC2: 0.91, *p* = 0.15) indicated that the selected basis dimensions were appropriate, and diagnostic plots confirmed a good model fit.

To determine the extent of plant thermal decoupling from air temperature at the microclimatic scale, we evaluated differences between plant and air temperature (AT) per species and hour using paired *t*-tests. In cases where normality assumptions were not met, specifically, *P. secunda* at P1 and *A. prolifera* at P2, we used the non-parametric Mann–Whitney U-test.

To test the importance of plant architectural traits in explaining thermal decoupling, we constructed a linear model including plant height, circularity index, porosity index, sampling date, and the interaction between height and circularity. In this linear model (LM), we included the interaction between height and circularity, as height represents the vertical dimension, and circularity reflects biomass distribution in three-dimensional space. Together, these variables provided an estimate of plant volume. Additionally, the species studied exhibited short stature and relatively consistent volume, enhancing the accuracy of this estimation. The linear model assumed a Gaussian distribution. Residuals were assessed for normality using the Shapiro–Wilk test (W = 0.959, *p* = 0.350) and homoscedasticity and linearity by inspecting residuals vs. fitted values plots, which showed no evident patterns or deviations. Additionally, we applied the Breusch–Pagan test (bptest() function from the lmtest package [141]), which indicated no significant heteroscedasticity (BP = 9.76, df = 5, *p* = 0.082). This result was supported by the non-constant variance score test (ncvTest() from the car package; [142]), which also showed no evidence of heteroscedasticity (χ^2^ = 0.54, df = 1, *p* = 0.461).

To assess interspecific differences in LT_50_, we performed a one-way ANOVA. Differences in LT_50_ between rosettes and shrubs growth forms were assessed using Mann-Whitney U-tests. Finally, to test the association between thermal decoupling and heat resistance, we used Spearman’s rank correlation between LT_50_ and the mean daytime thermal decoupling (TD) averaged per individual across the three measurement periods from 10:00 to 18:00.

## Figures and Tables

**Table 1:** Summary of the generalized additive model (GAM) explaining plant temperature as a function of the first two principal components (PC1 and PC2), derived from environmental variables. The model includes smooth terms for PC1 and PC2. Columns show the estimated degrees of freedom (edf), reference degrees of freedom (Ref.df) used for approximate F-tests, and associated *p*-values. Statistically significant *p*-values are shown in bold.

Effect	Edf	Red.df	F Value	*p*-Value
PC1	6.89	7.96	57.32	**<2.2 × 10^−16^**
PC2	1	1	2.54	0.114

**Table 2:** Results of the linear model testing the effects of plant architectural traits and sampling date on thermal decoupling (TD). The model included plant height (PH), circularity index (CI), porosity index (PI), and the interaction term PH × CI, with sampling date included as a fixed effect. For each term, degrees of freedom are shown as the numerator (numDF) and denominator (denDF), along with the sum of squares (Sum Sq), mean square (Mean Sq), F-value, *p*-value, and the proportion of explained variance (calculated as the ratio between the term’s sum of squares and the total sum of squares: 188.53). Statistically significant *p*-values are indicated in bold.

Effect	numDF	denDF	Sum Sq	Mean Sq	F Value	*p*-Value	Explained Variance
Height	1	21	45.02	45.02	18.8	**0.0003**	**23.88%**
Circularity index	1	21	36.21	36.21	15.12	**0.0008**	**19.21%**
Porosity index	1	21	2.57	2.57	1.07	0.3123	1.36%
Height and Circularity index interaction	1	21	7.00	7.00	2.93	0.1020	3.71%
Sampling date	1	21	47.44	47.44	19.82	**0.0002**	**25.17%**
Residuals	21	-	50.28	2.39			

## Data Availability

The datasets presented in this study can be found in the online repository: Mendeley Data, V1, https://doi.org/10.17632/ywpygww5fk.1. A preprint is available at https://doi.org/10.20944/preprints202506.1196.v1.

## Supplementary Materials

The following supporting information can be downloaded at: https://www.mdpi.com/article/10.3390/plants14132023/s1, Figure S1: Spearman rank correlation matrix for all environmental variables measured; Figure S2: Vapor pressure deficit during three measurement periods; Figure S3: Pearson’s correlation between thermal decoupling (TD, °C) and vapor pressure deficit (VPD, kPa); Figure S4: Macroclimate air (blue), microclimate air (green), and plant (red) temperatures were measured during three different periods; Figure S5: The relationship between temperature (°C) and heat damage (%). Table S1: Environmental variables measured at plant height in six alpine species from Nevados de Chillán. Table S2. Plant architectural traits for six alpine species from Nevados de Chillán.

## Abbreviations

The following abbreviations are used in this manuscript:

LT_50_	Lethal temperature that produces 50% tissue damage (°C)
TD	Thermal decoupling (°C)
WS	Wind speed (km h^−1^)
ST	Soil temperature (°C)
PAR	Photosynthetic active radiation (µmol m^−2^ s^−1^)
AT	Air temperature (°C)
RH	Relative humidity (%)
PCA	Principal component analysis
GAM	Generalized additive model
PT	Plant temperature (°C)
LM	Linear model
PH	Plant height (cm)
CI	Circularity index
PI	Porosity index
PGF	Plant growth form
IR	Infrared
Fv/Fm	The ratio of variable to maximum fluorescence in dark-adapted leaves
PhI	Photoinactivation (%)
VPD	Vapor pressure deficit (kPa)
