# Supplementary material for: Thermal Decoupling May Promote Cooling and Avoid Heat Stress in Alpine Plants
Source: Plants (Basel). 2025 Jul 2;14(13):2023. doi: 10.3390/plants14132023 (PMC12252459; doi:10.3390/plants14132023)
Supplement: Supplementary file 1 [file plants-14-02023-s001.zip › plants-3716405-supplementary.pdf]

SUPPLEMENTARY MATERIAL

Morales et al.,

FIGURES

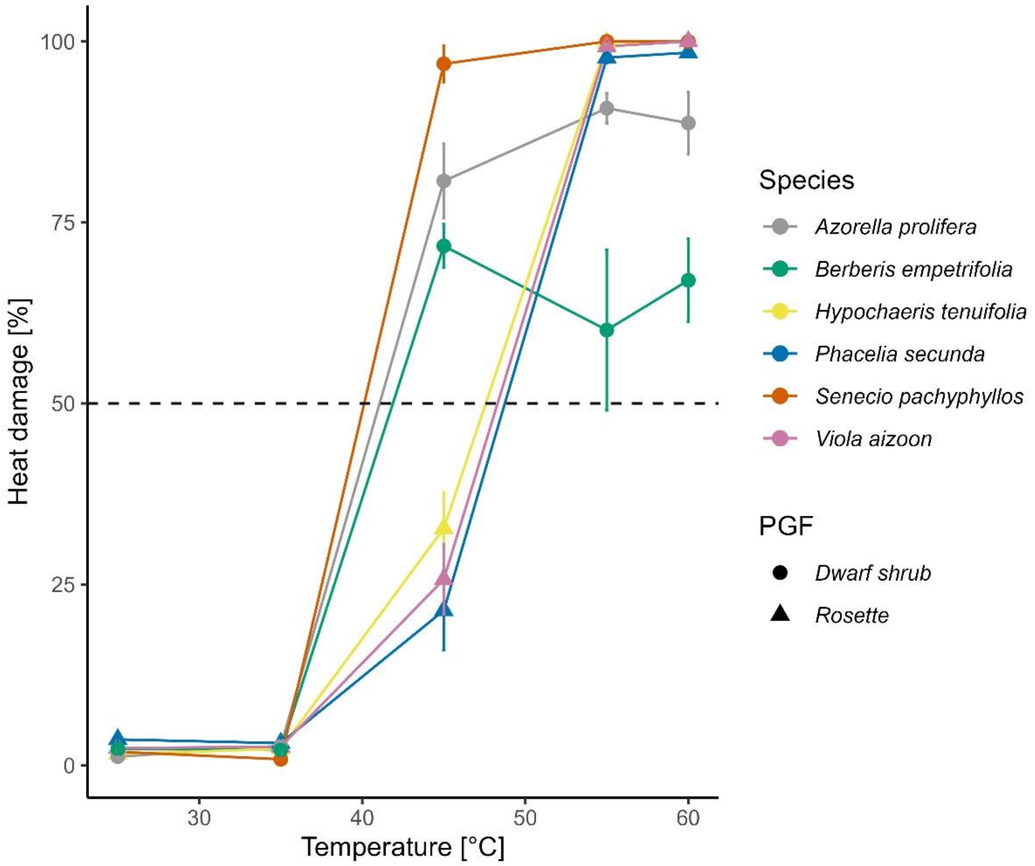

Figure S1. Relationship between temperature and heat damage.

The relationship between leaf temperature (°C) and percentage of heat-induced damage. Data points represent species means, with error bars indicating the standard error of the mean. Species are distinguished by color and shape, with shape denoting growth form. The dashed horizontal line denotes the 50% damage threshold (LT_{50}).

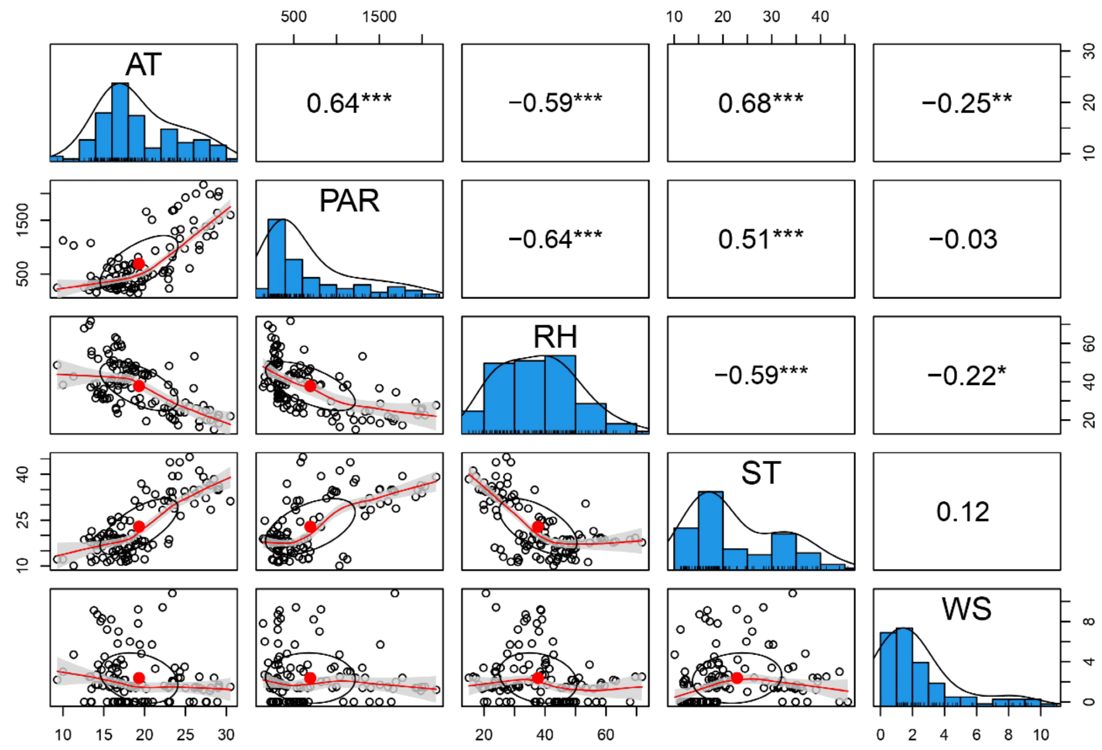

Figure S2. Correlation matrix of environmental variables

Spearman rank correlation matrix for all environmental variables measured. This includes air temperature (AT, °C), photosynthetically active radiation (PAR, μmol m^{-2} s^{-1}), relative humidity (RH, %), soil temperature (ST, °C), and wind speed (WS, km h^{-1}). The distribution of each variable is shown on the diagonal. Bivariate scatter plots with fitted lines are displayed at the bottom of the diagonal. Spearman's correlation coefficients are shown along with significance levels ($p < 0.05$ = *, $p < 0.01$ = **, $p < 0.001$ = ***).

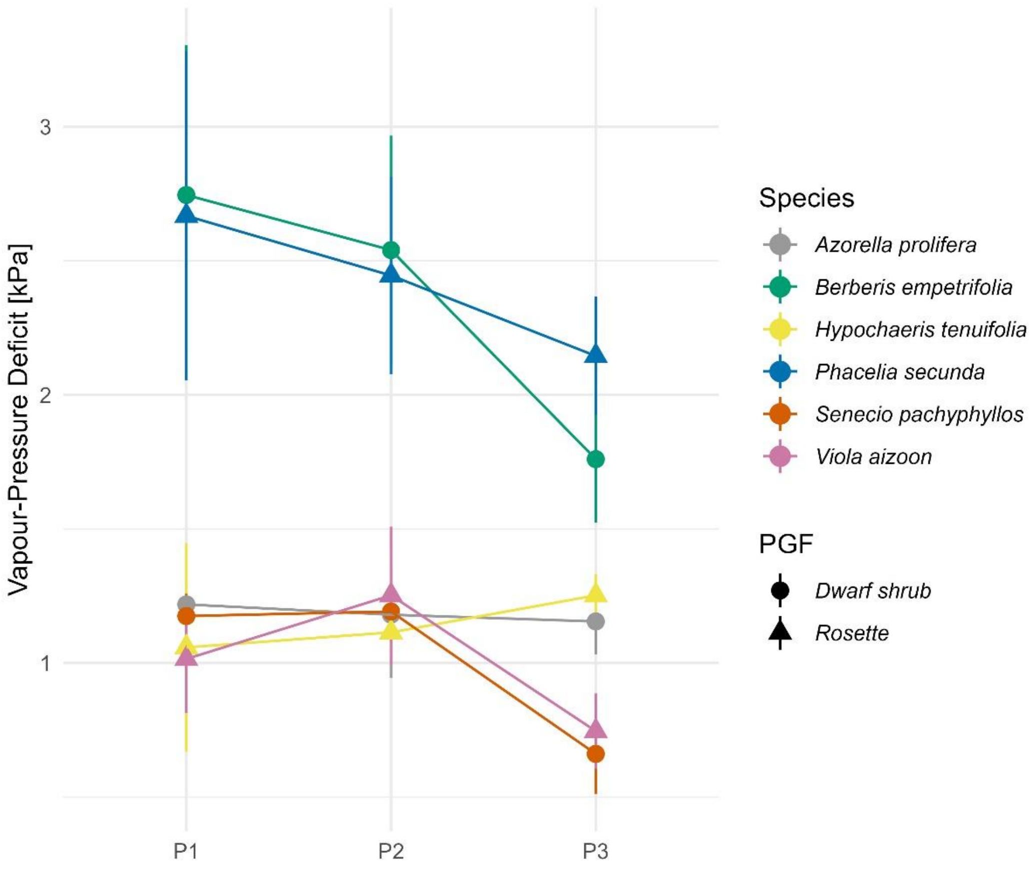

Figure S3. Vapor pressure deficit during the day

Vapor pressure deficit (VPD, kPa) during the three thermal measurement periods: P1 (10:00–12:00), P2 (13:00–15:00), and P3 (16:00–18:00). For *Berberis empetrifolia*, P1 corresponds to 12:00–13:00. Values represent means ± standard deviations. Colors denote species. Growth forms are indicated by shape: circles for dwarf shrubs and triangles for rosettes.

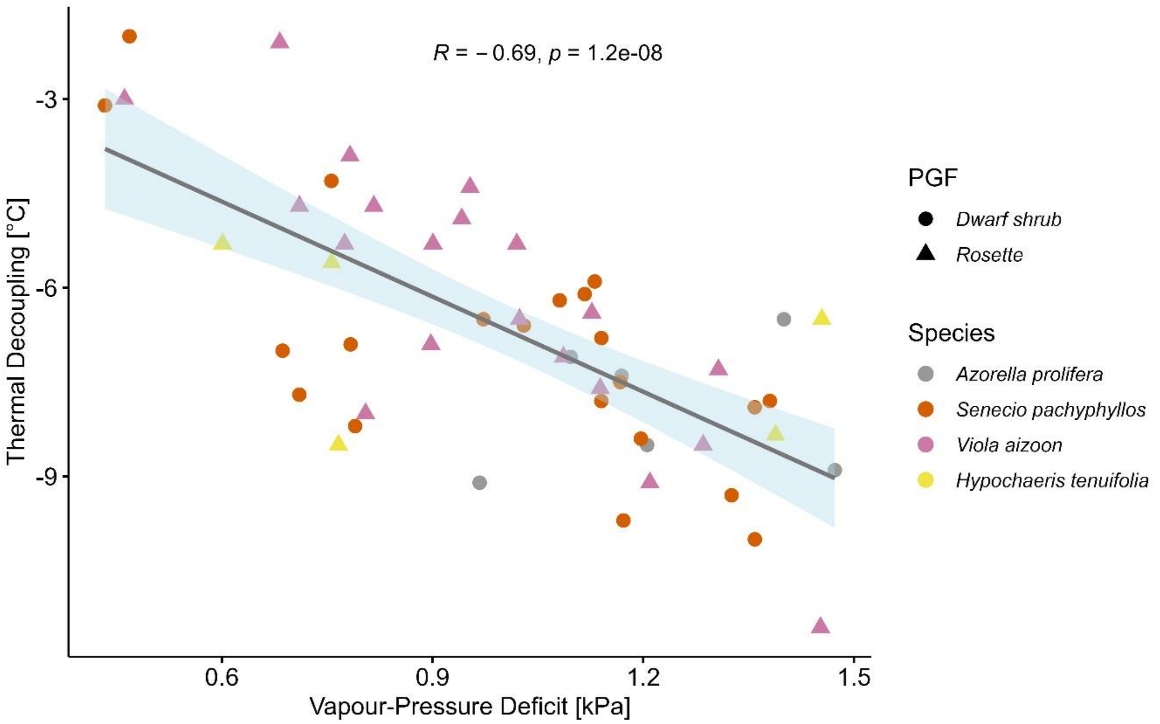

Figure S4. Correlation between thermal decoupling and VPD

Pearson's correlation between thermal decoupling (TD, °C) and vapor-pressure deficit (VPD, kPa). The correlation coefficient (r) and significance (p) are shown above each panel. Colors represent different species, while shapes denote growth forms: triangles indicate rosette species and circles indicate dwarf shrubs.

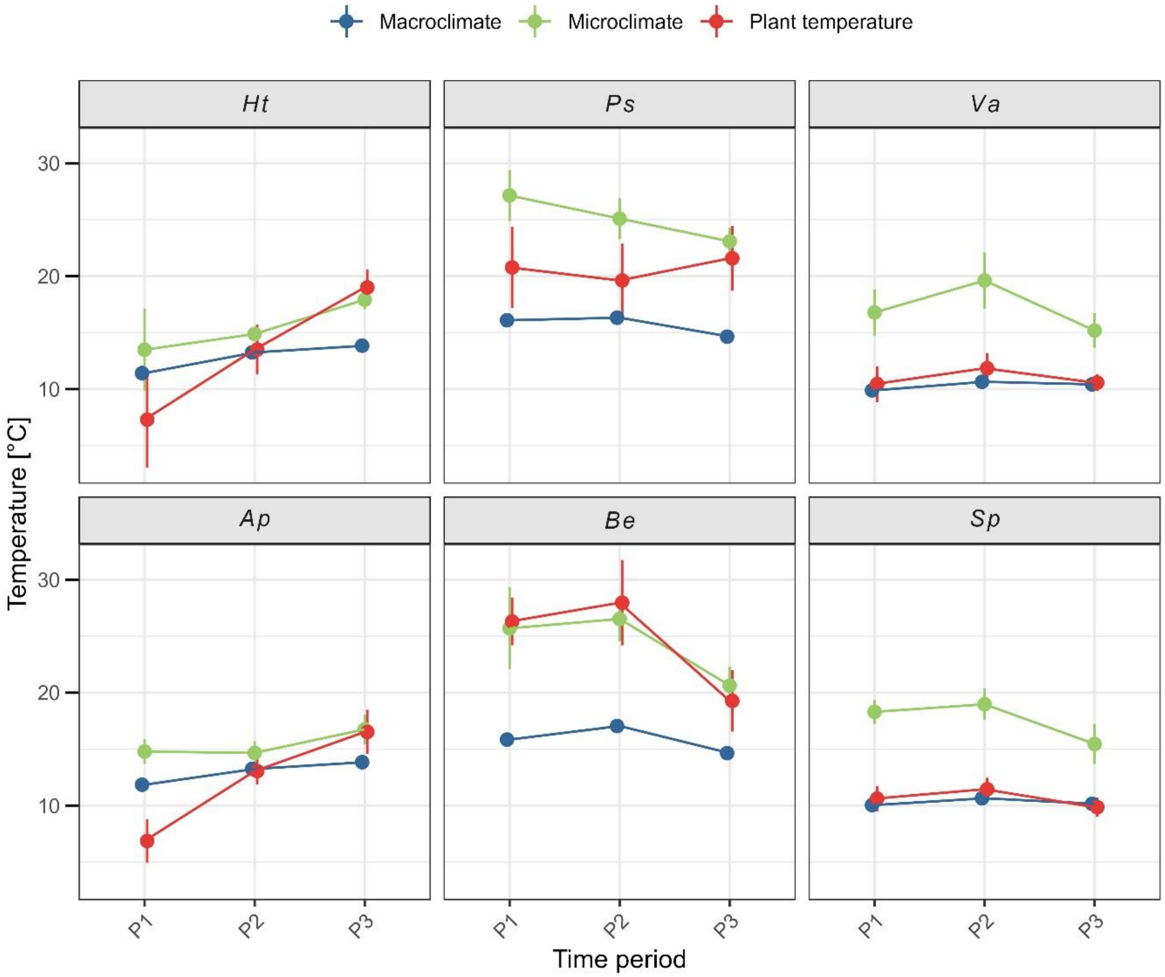

Figure S5. Macroclimate, microclimate, and plant temperatures.

Macroclimate air (blue), microclimate air (green), and plant (red) temperatures were measured during three periods: P1 (10:00–12:00), P2 (13:00–15:00), and P3 (16:00–18:00). For *Berberis empetrifolia*, P1 corresponds to 12:00–13:00. Species abbreviations correspond to rosette plants: Ht, *Hypochaeris tenuifolia*; Ps, *Phacelia secunda*; Va, *Viola aizoon*; and dwarf shrubs: Ap, *Azorella prolifera*; Be, *Berberis empetrifolia*; and Sp, *Senecio pachyphyllos*. Circles represent mean values with whiskers showing standard deviations. Blue bars indicate mean thermal decoupling (TD), calculated using macroclimate air temperature, with whiskers representing standard deviation. Macroclimate air temperatures (blue; °C) were obtained from the Termas de Chillán weather station (CN360042; 36°54′13″S, 71°24′36″W; 1708 m a.s.l.). As the station is located 270 m below the study site, air temperature was corrected assuming a lapse rate of −6.5 °C km^{-1} (Barry and Chorley, 1987; Frederick, 2008).

Table S1. **Environmental variables measured at plant height in six alpine species from Nevados de Chillán.**

Environmental variables include air temperature (AT, °C), soil temperature (ST, °C), relative humidity (RH, %), vapor pressure deficit (VPD, kPa), wind speed (WS, km h^{-1}), and photosynthetically active radiation (PAR, µmol m^{-2} s^{-1}), all measured at the species-specific plant height described in the table, along with their respective growth forms. Measurements were taken during three periods: P1 (10:00–12:00), P2 (13:00–15:00), and P3 (16:00–18:00). For *Berberis empetrifolia*, P1 corresponds to 12:00–13:00. Values are presented as mean ± standard deviation.

Growth form	Species	Date	Hour	Period	AT	ST	RH	VPD	WS	PAR
Rosette	*Hypochaeris tenuifolia*	March 8, 2023	10:51 ± 0:27	P1	13.5 ± 3.7	12.5 ± 2.0	34.8 ± 10.1	1.1 ± 0.4	2.4 ± 1.6	617.5 ± 379.0
Rosette	*Hypochaeris tenuifolia*	March 8, 2023	13:56 ± 0:11	P2	14.9 ± 0.5	18.6 ± 1.7	34.3 ± 3.0	1.1 ± 0.1	4.6 ± 3.0	311.5 ± 84.5
Rosette	*Hypochaeris tenuifolia*	March 8, 2023	16:06 ± 0:04	P3	17.9 ± 0.9	23.1 ± 2.7	39.1 ± 2.0	1.3 ± 0.1	4.8 ± 3.2	505.3 ± 290.9
Rosette	*Phacelia secunda*	March 7, 2023	11:09 ± 0:21	P1	27.4 ± 2.3	31.6 ± 2.9	25.6 ± 11.2	2.8 ± 0.6	1.4 ± 1.2	1440.7 ± 126.5
Rosette	*Phacelia secunda*	March 7, 2023	14:30 ± 0:09	P2	25.1 ± 1.8	40.8 ± 4.3	23.9 ± 3.8	2.4 ± 0.4	1.1 ± 1.3	885.8 ± 266.5
Rosette	*Phacelia secunda*	March 7, 2023	16:46 ± 0:05	P3	23.1 ± 1.2	33.3 ± 1.3	24.3 ± 3.3	2.2 ± 0.2	3.8 ± 3.5	1006.0 ± 366.3
Rosette	*Viola aizoon*	April 12, 2023	10:47± 0:22	P1	16.8 ± 2.1	12.8 ± 1.3	47.6 ± 4.1	1.0 ± 0.2	0.3 ± 0.8	378.0 ±125.2
Rosette	*Viola aizoon*	April 12, 2023	13:52 ± 0:12	P2	19.6 ± 2.5	17.9 ± 0.9	45.9 ± 3.7	1.3 ± 0.3	0.5 ± 0.9	257.7 ± 69.0
Rosette	*Viola aizoon*	April 12, 2023	16:18 ± 0:11	P3	15.2 ± 1.5	19.4 ± 1.2	57.1 ± 6.2	0.7 ± 0.1	2.2 ± 0.8	277.6 ± 44.6
Shrub	*Azorella prolifera*	March 8, 2023	10:57± 0:16	P1	14.8 ± 1.1	12.8 ± 1.4	27.8 ± 9.1	1.2 ± 0.2	3.8 ± 3.4	488.8 ± 294.3
Shrub	*Azorella prolifera*	March 8, 2023	13:56 ± 0:06	P2	14.7 ± 1.0	17.8 ± 1.8	29.3 ± 14.5	1.2 ± 0.2	5.6 ± 2.7	321.5 ± 15.5
Shrub	*Azorella prolifera*	March 8, 2023	16:02± 0:15	P3	16.7 ± 1.3	22.0 ± 2.7	39.7 ± 1.4	1.2 ± 0.1	5.0 ± 2.4	397.2 ± 225.9
Shrub	*Berberis empetrifolia*	March 7, 2023	12:59 ± 0:33	P1	25.7 ± 3.6	37.7 ± 2.8	18.1 ± 3.0	2.7 ± 0.6	2.0 ± 0.6	1528.0 ± 383.2
Shrub	*Berberis empetrifolia*	March 7, 2023	14:58 ± 0:06	P2	26.5 ± 2.0	34.5 ± 3.6	27.5 ± 4.1	2.5 ± 0.4	1.5 ± 0.3	1966.3 ± 155.4
Shrub	*Berberis empetrifolia*	March 7, 2023	17:01 ± 0:03	P3	20.6 ± 1.7	31.3 ± 3.0	28.1 ± 2.7	1.8 ± 0.2	4.8 ± 3.0	653.0 ± 423.6
Shrub	*Senecio pachyphyllos*	April 12, 2023	11:11± 0:11	P1	18.3 ± 1.1	13.5 ± 1.5	44.2 ± 1.7	1.2 ± 0.1	0.5 ± 0.9	577.1 ± 101.6
Shrub	*Senecio pachyphyllos*	April 12, 2023	14:06 ± 0:05	P2	19.0 ± 1.4	16.9 ± 1.0	46.0 ± 3.5	1.2 ± 0.2	0.6 ± 0.8	450.6 ± 136.1
Shrub	*Senecio pachyphyllos*	April 12, 2023	16:30± 0:06	P3	15.5 ± 1.8	18.2 ± 0.4	62.9 ± 5.3	0.7 ± 0.1	1.2 ± 1.2	349.7 ± 140.1

Table S2. Plant architectural traits for six alpine species from Nevados de Chillán.

Measured traits include growth form, plant height, circularity index, and porosity index. Values are presented as mean ± standard deviation.

Growth form	Species	Height [cm]	Circularity index	Porosity index
Rosette	*Hypochaeris tenuifolia*	6.7 ± 1.2	0.07 ± 0.02	0.30 ± 0.13
Rosette	*Phacelia secunda*	6.6 ± 1.5	0.37 ± 0.19	0.05 ± 0.02
Rosette	*Viola aizoon*	4.0 ± 0.7	0.20 ± 0.03	0.10 ± 0.07
Shrub	*Azorella prolifera*	10.8 ± 2.2	0.05 ± 0.02	0.16 ± 0.09
Shrub	*Berberis empetrifolia*	23.4 ± 8.9	0.05 ± 0.02	0.13 ± 0.05
Shrub	*Senecio pachyphyllos*	16.3 ± 6.0	0.25 ± 0.13	0.24 ± 0.09
